# One Step Preparation of Peptide-Coated Gold Nanoparticles with Tunable Size

**DOI:** 10.3390/ma12132107

**Published:** 2019-06-30

**Authors:** Yongmei Jia, Xiaoning Yan, Xin Guo, Guohua Zhou, Peilian Liu, Zhiguo Li

**Affiliations:** Key Laboratory of Clean Energy Materials Chemistry of Guangdong Higher Education Institutes, School of Chemistry and Chemical Engineering, Lingnan Normal University, Cunjin Road, Zhanjiang 524048, China

**Keywords:** CALNN, peptide, gold nanoparticle, self-assembling, size, tyrosine, trypsin

## Abstract

Gold nanoparticles (AuNPs) made from self-assembling peptides have been used in many research fields and attracted a great deal of attention due to their high stability, biocompatibility and functionality. However, existing preparation methods for peptide-coated AuNPs are post-synthesis processes, which are complicated and time consuming. Therefore, a one-step preparation method for peptide-coated AuNPs is proposed here. The AuNPs obtained by this method exhibit good stability. Importantly, peptide-coated AuNPs with precise different sizes can be prepared by this method through pH control of reducing reagent tyrosine in range of 10.0~12.7. Thus, the one-step preparation method proposed here provides a significant tool for the research in different fields concerning NP size, stability and biocompatibility.

## 1. Introduction

In recent decades, nanomaterials made from self-assembling peptides have attracted a great deal of attention from researchers because of the designability and functionality of peptides. Additionally, the components of such devices combine the features of Nature’s own nanodevices (proteins) such as specific recognition, biocompatibility, and cooperativity with the electronic, magnetic, and optical properties of nanomaterials [1]. Lots of nanomaterials with peptide-formed monolayers have been obtained, including silver [2,3], gold [4], carbon nanomaterials [5,6], magnetic nanoparticles [7,8] and silica nanoparticles [9]. These materials show advantages such as high biocompatibility and high stability in various applications, for example, as chemical sensors [10], biosensors [11], in enzyme activity analysis [12,13], bio-imaging [14], drug delivery [9], diagnosis and therapy [4,6,8] and nano-architecture fabrication [15,16]. However, most of these materials were prepared by covalent conjugation of peptides on the surface of nanomaterials. Compared with the self-assembly of peptides, covalent conjugation is complicated, difficult and uncontrollable [17]. Besides, the reagents and intermediate crosslinkers that are used for activation of the functional groups for conjugation, such as carbodiimide used to activate carboxylic acid groups for binding to free amines, usually cause nanoparticle aggregation [17]. In contrast, the self-assembly of peptides offers a number of advantages including bioorthogonality, control of biomolecule orientation, improved biomolecule valency, and fast self-assembly kinetics [17]. That is why many researchers have focused on the self-assembly of peptides.

**Scheme 1:**
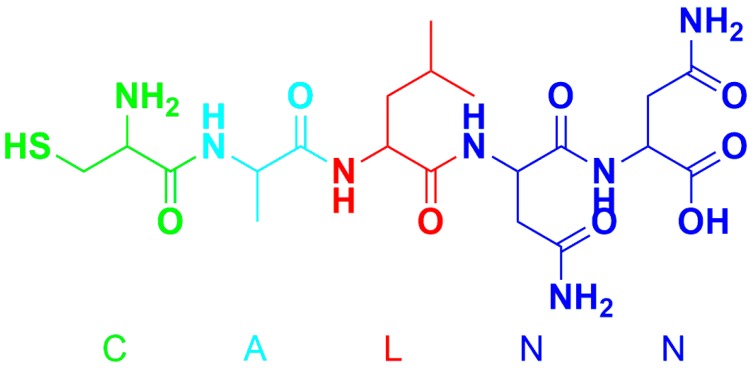
CALNN Structure.

In the past few years, peptides that are able to self-assemble on nanomaterials, for example, silver [18], gold [19,20,21], carbon nanotubes [22,23,24], magnetic nanoparticles [25], quantum dots [26] and MoS_2_ [27] have been identifed. Among these peptides, the pentapeptide CALNN (Scheme 1) that is able to form a dense self-assembled monolayer on gold nanoparticles (AuNPs) has attracted lots of attention [19]. As designed, the thiol group of cysteine (C) forms a covalent bond to the gold surface, while the alanine (A) and leucine (L) in positions 2 and 3 possess hydrophobic side chains that promote the self-assembly of the peptide, while the hydrophilic asparagine (N) and negatively charged terminal carboxylic acid group are pointing outwards, resulting in hydrophilic and negatively charged AuNPs [15,19]. Due to the remarkably easy preparation, high stability and biocompatibility of CALNN-coated AuNPs, they have been one of the most widely used biomaterials in recent years, greatly promoting the development of biosensors [28,29], disease diagnosis [30,31] and medicine [32,33]. Furthermore, the use of CALNN was extended to the stabilization and functionalization of quantum dots [34] and palladium nanoparticles [35] based on the coordination between metals and the sulfhydryl group of cysteine. However, the general protocol for preparation of peptide-coated AuNPs is a time consuming and post-synthesis process, which requires overnight incubation.

In consequence, a highly efficient preparation of peptide-coated nanomaterials is still a challenge. Previously, a one-step preparation of palladium nanoparticles was reported [35], suggesting the possibility of an improved preparation of peptide self-assembling nanomaterials. Based on that, this article reports a one-step preparation of peptide-coated gold nanoparticles with tunable particle size. To achieve the one-step preparation, tyrosine was used as reductant to reduce AuCl_4_^−^ in the presence of CALNN. The whole process can be done in 2 h, and the resulting self-assembled peptide AuNPs exhibit excellent stability over a wide range of pH values and salt concentrations. At the same time, the particle sizes of the AuNPs are accurately adjustable by control of the pH of the reaction buffer solution, which provides convenience and conditions for studies concerning the particle size of nanodevices, biosensors and other fields.

## 2. Materials and Methods

### 2.1. Materials

Sodium hydroxide, gold(III) chloride trihydrate and tyrosine were purchased from Shanghai Macklin Biochemical Co., Ltd. (Shanghai, China). Peptides (CALNN and CALNNGGARK_(FITC)_) were synthesized and purified by ChinaPeptides Co., Ltd. (Shanghai, China). The Bicinchoninic Acid (BCA) Protein Assay Kit was obtained from Sangon Biotech (Shanghai) Co., Ltd. (Shanghai, China). All reagents used in research were analytically pure and used without further purification. Ultrapure water with 18.2 MΩ resistance was freshly prepared by a laboratory water purification system (Master-D; Hitech Instruments, Shanghai, China) every day.

### 2.2. General Protocol for One-Step Preparation

CALNN and chloroauric acid were dissolved in ultrapure water to form 1 mmol/L and 1% solutions, respectively. Tyrosine was dissolved in 0.05 mol/L NaOH or alkaline Britton-Robison (BR) buffer solution (0.04 mol/L). The preparation of peptide-coated AuNPs was as follows:

Method A: 2 mL of tyrosine was mixed with 0.9 mL CALNN, and then 0.1 mL chloroauric acid was added, quickly mixed and reacted at 37 °C for 2 h with gentle rotation.
Method B: 2 mL of tyrosine was mixed with 0.1 mL chloroauric acid, and then 0.9 mL CALNN was added, and the mixture reacted at 37 °C for 2 h with gentle rotation.
Method C: 2 mL of tyrosine was added to the mixture of 0.9 mL CALNN and 0.1 mL chloroauric acid, mixed well and reacted at 37 °C for 2 h with gentle rotation.

All of the reaction mixtures were centrifuged at 17,800× *g* for 20 min then centrifugal washing with ultrapure water was repeated four times to remove any unreacted starting materials. The obtained peptide-coated AuNPs were stored at 4 °C.

### 2.3. Preparation of CALNN-Coated AuNPs with Different Size

The CALNN-coated AuNPs with different sizes were prepared by replacing tyrosine dissolved in NaOH to that dissolved in BR buffer solution with different pH. The preparation process was as described in Method C in Section 2.2.

### 2.4. Preparation of Functional AuNPs

CALNN and CALNNGGARK_(FITC)_ were dissolved in ultrapure water to give 1 mmol/L solutions, respectively. A mixed peptide solution was prepared by mixing CALNN and CALNNGGARK_(FITC)_ with a volume ratio of 9:1, and then 0.9 mL of peptide solution was mixed with 0.1 mL chloroauric acid. Following that, 2 mL of tyrosine was added and reacted following the general protocol described in Section 2.2.

### 2.5. Determination of Trypsin

In a general process for trypsin detection, functional AuNPs properly diluted with Tris-HCl buffer solution were mixed with a certain amount of trypsin. The mixture was incubated at 37 °C for 100 min with a rotatory mixing. After incubation, the fluorescence of the solution was measured.

### 2.6. Characterization

#### 2.6.1. Transmission Electron Microscopy (TEM)

The samples for TEM were prepared by placing a drop of the colloidal dispersion of the CALNN-coated AuNPs onto a carbon-coated copper grid followed by natural evaporation of the solvent. A HT7700 120 kV Compact-Digital microscope (Hitachi, Tokyo, Japan) at 100 kV was used for measurements.

#### 2.6.2. UV–Visible Absorption Spectroscopy

The UV–vis absorption spectra of AuNPs were measured with an Infinite M200 Pro spectrometer (Tecan, Männedorf, Zürich, Switzerland).

#### 2.6.3. Thermogravimetric Analysis (TGA)

The samples for TGA were concentrated by centrifugation and the supernatant removed, followed by freeze drying. The TGA was performed on a Pyris 1 system (PerkinElmer, Waltham, MA, USA).

#### 2.6.4. Dynamic Light Scattering (DLS)

The particle size, polymer dispersity index, and zeta potential data of the AuNPs were measured with a 90Plus PALS system (Brookhaven Instruments, Holtsville, NY, USA).

#### 2.6.5. Fluorescence Spectroscopy

The fluorescence measurements for the determination of trypsin were performed on a Tecan Infinite M200 Pro instrument.

#### 2.6.6. Energy dispersive X-ray spectroscopy (EDS)

High angle annular dark field (HAADF) TEM images and TEM-energy dispersive X-ray spectroscopy (EDS) mapping of samples are obtained from Tecnai G2 F30 (FEI, Hillsboro, OR, USA).

## 3. Results and Discussion

### 3.1. AuNP Preparation

**Figure 1:**
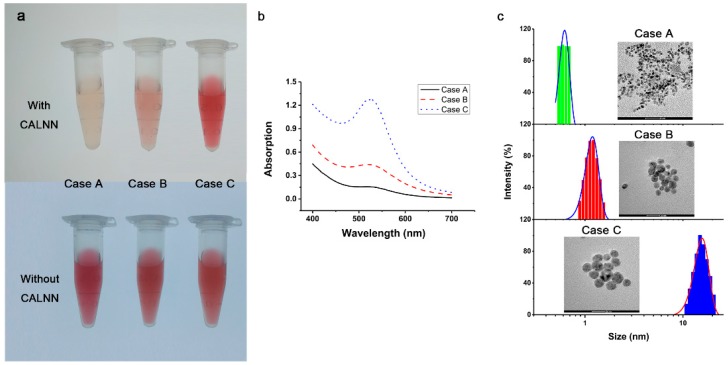
(**a**) Photographs of the products obtained by different preparation procedures (upper) with and (lower) without CALNN. (**b**) UV–vis absorption spectra corresponding to the products with CALNN. (**c**) TEM and DLS analysis of the size of AuNPs in different routes. Method A: AuCl_4_^−^ was adding to the mixture of tyrosine and CALNN; Method B: AuCl_4_^−^ and tyrosine was mixed firstly and followed by CALNN; Method C: tyrosine was adding to the mixed solution of AuCl_4_^−^ and CALNN.

To dissolve tyrosine in aqueous solution and ensure its reducibility, tyrosine was prepared by dissolving in 0.05 mol/L NaOH solution, because the reducibility of the phenol group of tyrosine is related to the pH of the solution [36]. In a preliminary experiment, three different procedures described in the Materials and Methods section were investigated, varying the feeding order of reactants. The results are shown in Figure 1. Firstly, the preparation of AuNPs was conducted by the addition of AuCl_4_^−^ into the mixture of tyrosine and peptide (referred to as Method A). As shown in Figure 1a, the color of the reactant mixture became a little orange after 2 h of incubation. Secondly, the preparation was conducted by the addition of peptide into the mixture of AuCl_4_^−^ and tyrosine (referred to as Methos B). After incubation, the color of mixed solution only turned to light pink after 2 h (Figure 1a). In contrast, when the preparation was conducted by the addition of tyrosine into the mixture of AuCl_4_^−^ and peptide (referred to as Method C), the color of the mixture turned wine red, the typical color of AuNPs, after incubation, indicating the formation of AuNPs.

**Figure 2:**
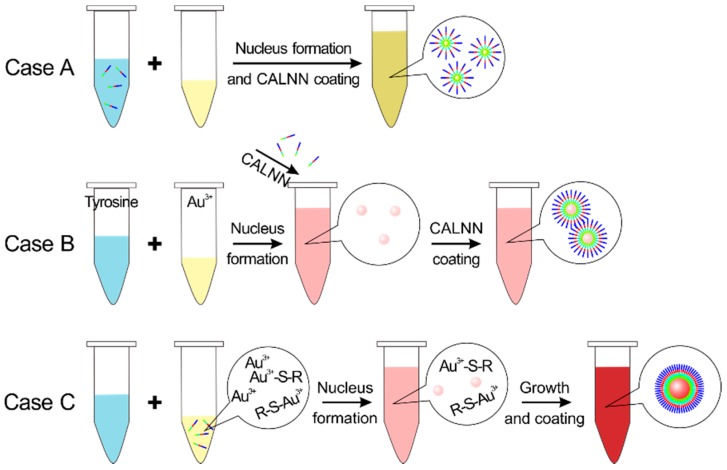
Schematic illustration of the formation of AuNPs by the three different routes.

In order to investigate the above procedure, UV–vis spectroscopy was employed to characterize the resulting solutions. As can be seen from the Figure 1b, an obvious and strong absorption is observed at about 520 nm for Method C, which can be ascribed to the surface plasma resonance (SPR) absorption of AuNPs [37,38]. On the contrary, only weak absorption peaks for methods A and B. These results indicate that the formation of AuNPs is closely related to the feeding order of reactants. By calculating the ratio between the absorption of SPR (A_SPR_) and 450 nm (A_450_) according to [37], AuNPs in different size were obtained from the three different routes. Method A AuNPs have the smallest size (A_SPR_/A_450_ = 0.8), while those produce by Method C are the largest (A_SPR_/A_450_ = 1.3). To further confirm the sizes of the AuNPs, TEM and DLS analyses of the AuNPs obtained by the different routes were performed (Figure 1c). The results show the dimensional relations between AuNPs are consistent with the UV–vis absorption data. Additionally, the successful preparation of AuNPs without CALNN by the three different methods (Figure 1a, lower) indicates that the formation of AuNPs is related to the addition of CALNN. Based on that, the main cause of these phenomena can be presumably described as follows; As shown in Figure 2, although tyrosine mixed with CALNN firstly reacts with peptide (Figure S1) in Method A, decreasing the concentration of tyrosine, the excess tyrosine still reduces Au^3+^ to form gold nuclei. More importantly, small gold nuclei were coated by CALNN as soon as formed, which restricts the nuclei growth. In Method B, small gold nuclei were formed when AuCl_4_^−^ was mixed with tyrosine, and then grew a little bit in the minutes previous to the addition of CALNN, as the color of mixture was changed to light pink. Then, the CALNN coated the small AuNPs when the peptide was added, and restricts the growth of the AuNPs. As a consequence, the particle size of the gold nanoparticles is relatively larger than in Method A. Only Method C ensures the preparation of large gold nanoparticles, because the sulfhydryl group of the cysteine residue of the CALNN peptide will form a chelate with Au^3+^ [39] and is thus unable to assemble on the AuNP surface. Therefore, the remaining free gold ions are able to nucleate and grow to form large AuNPs. After consumption of the free gold ions, the chelated Au^3+^ is absorbed onto the nanoparticles and reduced at a low reaction rate, because of the low activity and limited diffusion of ions, cooperating with the coating of CALNN. As a result of that, final AuNPs with desired size was obtained. This procedure was described as diffusion limited formation of AuNPs in [40].

### 3.2. Optimization

**Figure 3:**
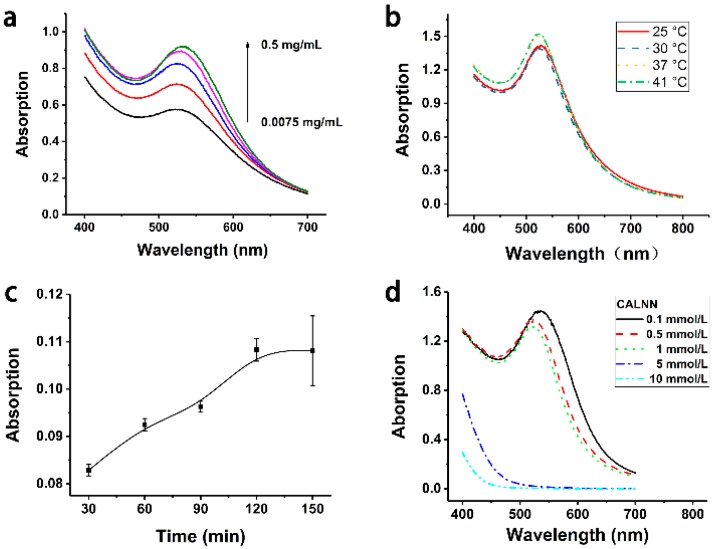
(**a**) UV–vis absorption spectra of the product prepared with different concentrations of tyrosine or (**b**) at different temperature. (**c**) The absorption of AuNPs at 520 nm changes with the reaction time. (**d**) UV–vis absorption spectra of AuNPs prepared with different concentrations of CALNN.

To improve the preparation efficiency, the experimental conditions for the synthesis of CALNN-coated AuNPs were investigated. As we know, the reducing efficiency of a reactant depends on its concentration. Thus, the concentration of tyrosine in the preparation was studied first. Considering the solubility of tyrosine in water is 0.45 mg/mL and slightly increased in alkaline solutions, the tyrosine concentrations in the tests was set in the range of 0.0075–0.5 mg/mL, with a AuCl_4_^−^ concentration of 3 mmol/L. Figure 3a shows the UV–vis absorption spectra of AuNPs obtained with different tyrosine concentrations. As can been seen, with the increase of tyrosine concentration, the SPR absorption peak of AuNPs at 520 nm was enhanced, indicating an increased yield and quality of AuNPs with increasing tyrosine concentration. Therefore, the optimal condition is set at the concentration of 0.5 mg/mL to ensure the solubility and reducing ability of tyrosine.

Since peptides were employed in the preparation process, the ambient temperature for preparation should be moderate enough to maintain the activity and stability of peptides. On the other hand, the reduction reaction is temperature dependent. Therefore, the temperature for preparation was investigated in the range of 25–41 °C. It can be seen from the UV–vis absorption spectra of AuNPs in Figure 3b that AuNPs can be generated within this range of temperatures with little difference in the SPR absorption intensity. Higher temperatures result in higher absorption intensity and the spectra for 37 °C and 41 °C are identical, indicating that temperatures higher than 37 °C have no significant effect on the preparation of AuNPs, so 37 °C was chosen as the better choice for the reaction.

Reaction time is another important condition should be considered, so the SPR absorption intensity of the reaction mixture at 520 nm was measured at different times during the reaction process. The results show that AuNPs were generated after a reaction time of 30 min, as the color of solution changed to pink and the SPR absorption of the diluted solution at 520 nm was 0.08. The SPR absorption of AuNPs gradually increased with time within 30–120 min, and then there was no significant change at 120–150 min, indicating that the reduction reaction was complete. Therefore, the optimal reaction time for peptide-coated AuNP preparation is at least 120 min.

In addition, the effect of concentration of CALNN on the preparation of AuNPs was investigated as well, since the presence of CALNN is closely related to the formation of AuNPs (Section 3.1). It can be seen from Figure 3d that, concentrations of CALNN in the range of 0.1~1 mmol/L have little influence on the preparation of AuNPs. DLS analysis (Figure S2) indicated that these AuNPs have similar sizes, but 1 mmol/L CALNN provides the best monodispersity of AuNPs (polymer dispersity index, PDI = 0.19). Higher concentrations of CALNN restrict the formation of large AuNPs, because all of the Au^3+^ formed chelates with CALNN. As a result, the gold nucleus formation and CALNN coating happened simultaneously, forming AuNPs smaller than 1 nm as the DLS measurements show. Consequently, 1 mmol/L CALNN was selected for the optimized preparation of peptide-coated AuNPs.

### 3.3. Characterization

**Figure 4:**
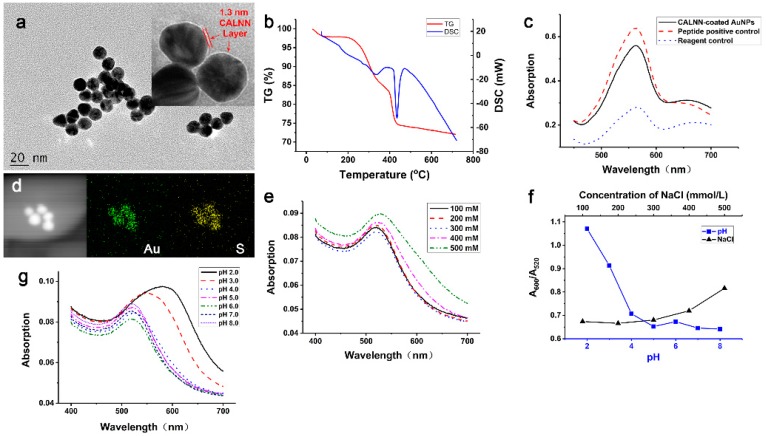
(**a**) TEM image of CALNN-coated AuNPs prepared under optimized conditions. Inset: negative staining TEM of AuNPs showing the surface coating. (**b**) The TG curve and corresponding DSC curve of CALNN-coated AuNPs. (**c**) UV–vis absorption spectra of the BCA solution reacted with CALNN-coated AuNPs and CALNN positive control. (**d**) TEM-HAADF image and TEM-EDS mapping of CALNN-coated AuNPs. (**e**) UV–vis absorption spectra of obtained CALNN-coated AuNPs with different concentration of NaCl. (**f**) The corresponding change of aggregation factor (A_600_/A_520_) to concentration of NaCl and different pH values. (**g**) UV–vis absorption spectra of CALNN-coated AuNPs at different pH.

TEM was employed to characterize the obtained AuNPs. As shown in Figure 4a, the AuNPs are well dispersed with uniform spherical shape, and about 15 nm in size. Additionally, a 1.3 nm thin layer was observed in the negative staining TEM image (Figure 4a inset). This layer is believed to be the CALNN cover, because the thickness of 1.3 nm is comparable to the 1.6 nm length of CALNN obtained from molecular models [15]. The weight loss (about 23%) of AuNPs in the thermogravimetric (TG) analysis is also evidence of coating on the surface. As shown in Figure 4b, excluding the small weight loss (<3%) at T < 100 °C that due to the moisture of sample, the CALNN-coated AuNPs display a two-stage decomposition beginning at 200 °C and 385 °C, respectively, corresponding to two exothermic peaks at 309 °C and 411 °C in the differential scanning calorimetry (DSC) curve. To further confirm the CALNN cover, the peptide ligands coated on the AuNPs were analyzed by the BCA method, which is a general method for the determination of peptides and proteins. It can be seen from Figure 4c that the UV–vis absorption spectrum of the reagent control solution displays a low intensity at 562 nm. When the peptides are present, the peptide bond can reduce Cu^2+^ to form Cu^+^ under alkaline conditions. Then Cu^+^ will form coordination compounds with bicinchoninic acid, resulting in the enhancement of the absorption at 562 nm. Correspondingly, the obtained AuNPs in this research also show an absorption change like that of a peptide control, indicating that the AuNPs were coated with peptide. Additionally, high angle annular dark field (HAADF) TEM images and TEM-energy dispersive X-ray spectroscopy (EDS) mapping of CALNN-coated AuNPs display strong and uniformly distributed S signals along with Au signals (Figure 4d). The S element must come from the sulfhydryl group of cysteine residues. Accordingly, the AuNPs are evidently densely coated by CALNN peptide.

The stability of the obtained AuNPs was also investigated. As claimed in [19], the dense cover of CALNN and the negative charged carboxylic acid groups on the surface are the reasons for the high stability of AuNPs. Zeta potential measurement revealed that the as-prepared AuNPs possess a zeta potential of –14.1 mV under alkaline conditions (pH 7.4). On the basis of that, the investigation shows that CALNN-coated AuNPs prepared by the one-step method described here exhibit high stability against ion strength and pH, as expected. The UV–vis absorption spectra of AuNPs in different concentrations of NaCl ranging from 100 mmol/L to 300 mmol/L are identical. When the concentration of NaCl increases to 400 mmol/L and 500 mmol/L, the absorptions at wavelengths larger than 600 nm are enhanced dramatically with the color change of solutions (Figure 4e), indicating the aggregation of AuNPs [19]. When the ratio between the absorption at 600 nm (A_600_) and 520 nm (A_520_) is plotted as the aggregation factor for the NaCl concentration (Figure 4f), it is clear that the AuNPs are capable of bearing ion strengths as high as 300 mmol/L NaCl. Similar results showing the stability of AuNPs against pH are displayed in Figure 4f,g, which indicate the AuNPs is stable at pHs higher than 4.0. These results demonstrate that the CALNN-coated AuNPs prepared by the one-step method described in this paper possess high stability, enough to be stable over a wide range of pH and salt concentrations as reported in [19]. The high stability also ensures the long-term storage of AuNPs, indicated by the fact that the UV–vis absorption spectra of prepared AuNPs stored at 4 °C over 6 months did not exhibit any obvious changes (Figure S3).

### 3.4. Size Control

**Figure 5:**
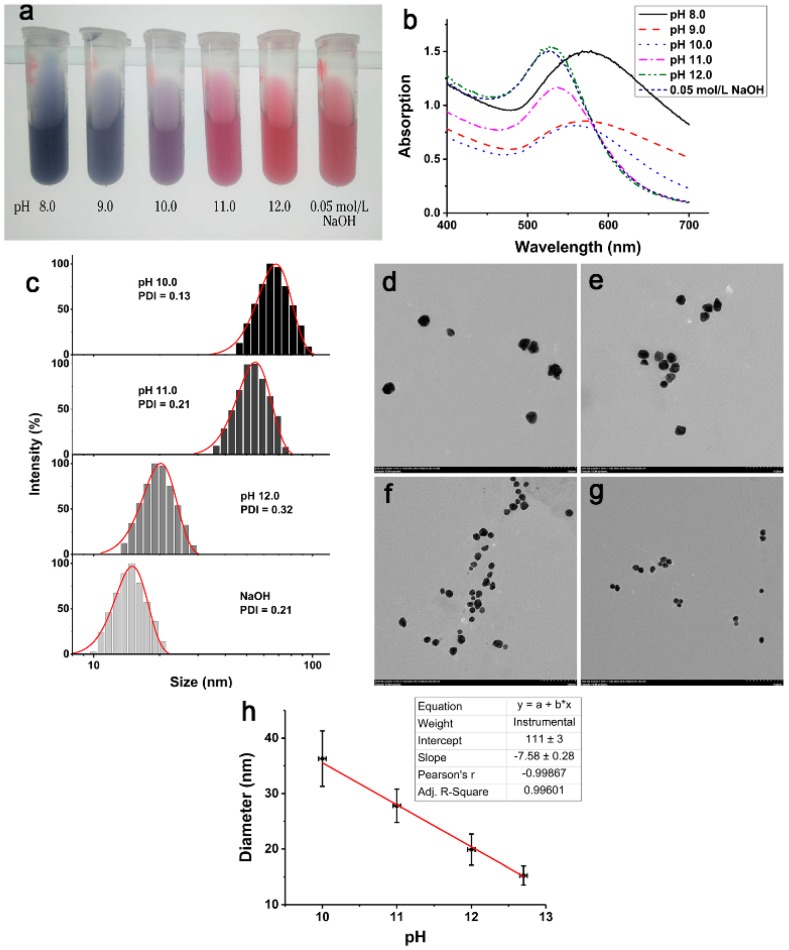
Picture of the products prepared by dissolving tyrosine at different pHs (**a**) and the corresponding UV–vis absorption spectra (**b**). (**c**) DLS and (**d**–**g**) TEM images of CALNN-coated AuNPs obtained at pH 10.0, 11.0 12.0 and 0.05 mol/L NaOH, respectively. (**h**) Relationship between the size and pH of tyrosine solutions.

As we know, the reducibility of the phenol group of tyrosine is related to the alkalinity. Thus, it was expecting that the size of peptide-coated AuNPs could be controlled by varying the pH of the solution to adjust the reducing ability of tyrosine. To verify this idea, BR buffer solution was chosen to maintain the pH of reaction mixture as well as 0.05 mol/L NaOH. As shown in Figure 5a, the color of AuNPs obtained at different pHs changes from blue to red corresponding to pH values from 8.0 to 12.7 (calculated from 0.05 mol/L NaOH). Correspondingly, the SPR absorption of AuNPs exhibited a red shift with pH decrease (Figure 5b).

It is well known that the color and SPR absorption of AuNPs are related to the size of the AuNPs [37,38]. To further confirm the size of AuNPs, DLS and TEM analyses were performed. The AuNPs obtained at pH 8.0 and 9.0 appear to be unstable and agglomerate, probably due to the fact that the size of the AuNPs is too large for the CALNN ligand. On the other hand, AuNPs obtained in the pH range of 10.0 to 12.7 are well dispersed with a PDI of less than 0.32 and the display pH-related sizes as expected (Figure 5c–g). Furthermore, when the size of AuNPs measured from TEM images was plotted versus pH, a linear relationship was obtained (Figure 5h). As shown in Figure 5h, the size of AuNPs decreases linearly with the increase of pH in the range from 10.0 to 12.7, with slope = −7.58 ± 0.28, intercept = 111 ± 3, and the coefficient of determination R^2^ reaching 0.996. As a consequence, the size of AuNPs can be controlled precisely in the range of 15~35 nm according to the linear equation. That is different from and demonstrates the advantages of the method proposed here over the general protocol for controlling the size of metal NPs, which is usually done by changing the concentration of reducing reagents. Therefore, the method for one-step preparation of peptide-coated AuNPs proposed here not only provides a convenient route to produce nanoparticles, but also provides a tool for metal NP size-related studies and applications.

### 3.5. Application

**Figure 6:**
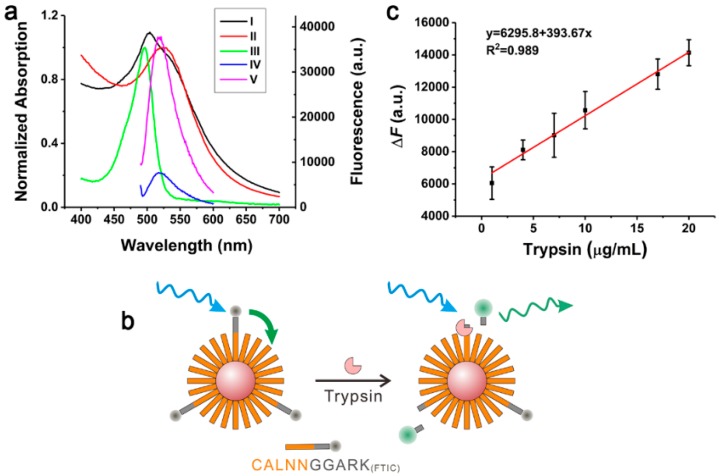
(**a**) Normalized UV–vis absorption spectra of (I) functional AuNPs that labeled with FITC, (II) CALNN-coated AuNPs, (III) FITC, and the (IV) fluorescence spectra of functional AuNPs without and (V) with trypsin. (**b**) Scheme of the fluorescence-based trypsin detection with functional AuNPs. (**c**) The standard curve for trypsin detection with functional AuNPs.

To embody the application of one-step prepared peptide-coated AuNPs, a functional peptide (CALNNGGARK_(FITC)_) composed of CALNN, substrate peptide (GGARK) for trypsin and fluorophore fluorescein isothiocyanate (FITC) was employed to prepare functional AuNPs. This preparation can be easily achieved by mixing functional peptide with CALNN in a 1:9 molar ratio during the preparation process. As shown in Figure 6a, compared with the UV–vis absorption spectrum of CALNN-coated AuNPs (curve II), the absorption of functional AuNPs (curve I) at about 490 nm obviously enhanced, attributing to the absorption of FITC (curve III). Consequently, functional AuNPs specific for trypsin were confirmed. Since the fluorescence spectrum of FITC (curve V) and UV–vis absorption spectrum of AuNPs (curve II) overlap completely, AuNPs are an excellent fluorescence energy receptor for FITC. Thus, the fluorescence of FITC linked to AuNPs by functional peptide is quenched through fluorescence resonance energy transfer (FRET) (Figure 6a, curve IV). In the presence of trypsin, the substrate region of the functional peptide will be cleaved between the arginine (R) and lysine (K), resulting in the separation of FITC from AuNPs (Figure 6b), and then the recovery of fluorescence (Figure 6a, curve V). Accordingly, the detection of trypsin can be realized. As shown in Figure 6c, the fluorescence was measured after adding trypsin in different concentrations and a good linear relationship between the concentration and fluorescence was obtained in the range of 1–20 μg/mL, with a LOD of 0.5 μg/mL (3σ). These results fully prove that the peptide-coated AuNPs prepared in this research have the advantage of easy functionalization and could be used for the fluorescence determination of enzymes.

## 4. Conclusions

In summary, a one-step method for the preparation of peptide-coated and functionalized AuNPs is proposed here. This preparation is based on the reduction of AuCl_4_^−^ by tyrosine under alkaline conditions. The optimal conditions for the preparation, such as mass of reactants, reaction time and temperature have been investigated. Under the optimized conditions, CALNN-coated AuNPs with a size of 15 nm have been obtained, which exhibit excellent stability against ion strength and pH. Importantly, AuNPs of different sizes ranging from 15 nm to 35 nm can be obtained by adjusting the pH of tyrosine in the range of 10.0–12.7. The size of the AuNPs shows a good linear relationship with the pH, with a high coefficient of determination R^2^ of 0.996. Accordingly, peptide-coated AuNPs of 15~35 nm size can be accurately controlled by adjusting the pH of tyrosine. Finally, the peptide-coated AuNPs can be easily functionalized by adding a functional peptide in the preparation process. For example, CALNNGGARK_(FITC)_ functionalized peptide-coated AuNPs have been obtained for trypsin determination in range of 1–20 μg/mL. Consequently, the presented green and convenient one-step preparation of peptide-coated AuNPs with continuous and precise controlled size provides a significant tool for the study of self-assembly, nanodevices, bioanalysis and other research fields concerning NPs size, stability and biocompatibility.

## Supplementary Materials

The following are available online at https://www.mdpi.com/1996-1944/12/13/2107/s1, Figure S1: Fluorescence spectra of tyrosine before and after addition of CALNN, Figure S2: DLS analysis of AuNPs prepared with different concentrations of CALNN, Figure S3: UV–vis absorption spectra of as prepared CALNN-coated AuNPs and the AuNPs after 6 months storage at 4 °C.
