# Supplementary material for: One Step Preparation of Peptide-Coated Gold Nanoparticles with Tunable Size
Source: Materials (Basel). 2019 Jun 30;12(13):2107. doi: 10.3390/ma12132107 (PMC6651442; doi:10.3390/ma12132107)
Supplement: Supplementary file 1 [file materials-12-02107-s001.pdf]

Supplementary Materials

One step preparation of peptide-coated gold nanoparticle with tunable size

Yongmei Jia, Xiaoning Yan, Xin Guo, Guohua Zhou*, Peilian Liu and Zhiguo Li

School of Chemistry and Chemical Engineering, Key Laboratory of Clean Energy Materials Chemistry of Guangdong Higher Education Institutes, Lingnan Normal University, Cunjin Road, Zhanjiang, Guangdong 524048, China.

* Correspondence: ghzhou@lingnan.edu.cn; Tel.: +86-759-3183947

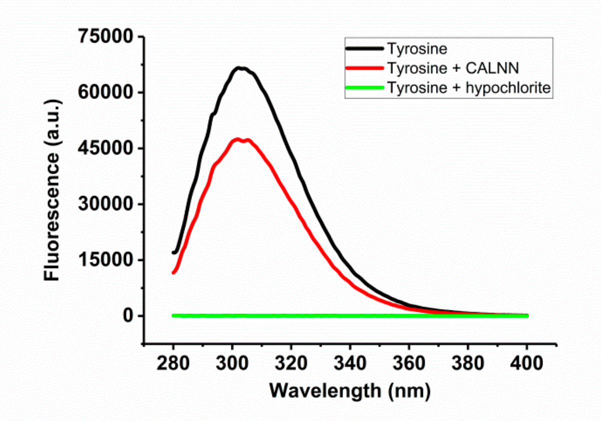

Figure S1. Fluorescence spectra of tyrosine before and after addition of CALNN. The spectrum of tyrosine with the addition of sodium hypochlorite was used as positive control. The fluorescence were measured with excitation at 230 nm.

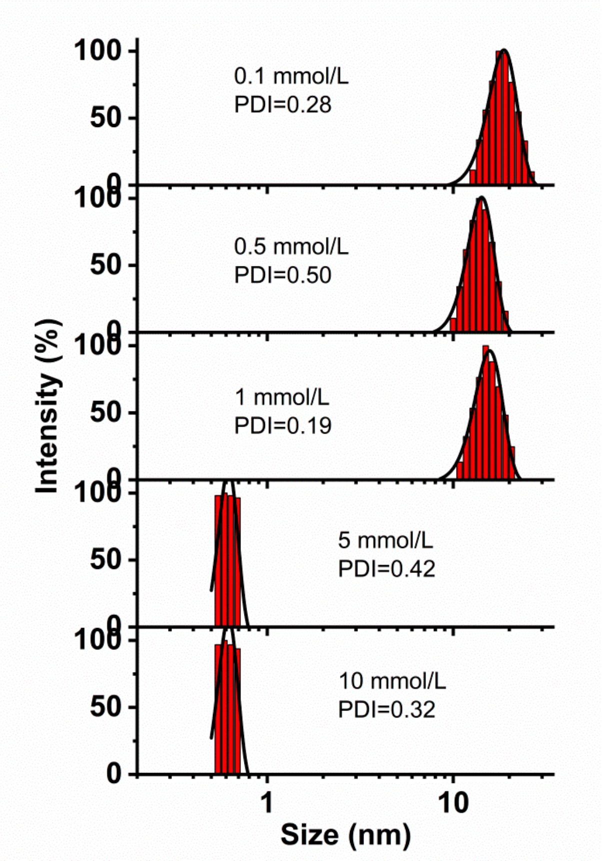

Figure S2. DLS analysis of AuNPs prepared with different concentrations of CALNN.

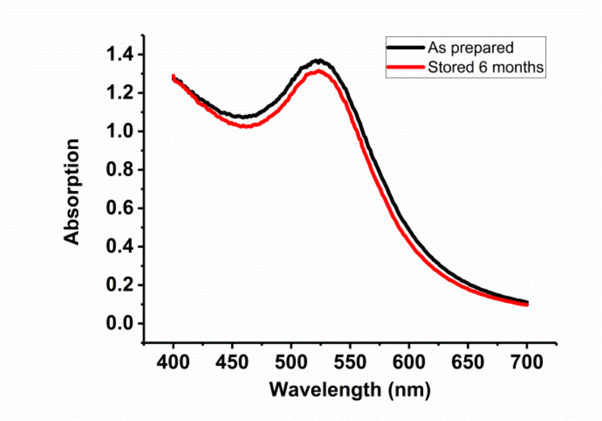

Figure S3. UV-vis absorption spectra of as prepared CALNN-coated AuNPs and the AuNPs after 6 months storage at 4 ℃.
